# Circ-KIAA0907 inhibits the progression of oral squamous cell carcinoma by regulating the miR-96-5p/UNC13C axis

**DOI:** 10.1186/s12957-021-02184-8

**Published:** 2021-03-14

**Authors:** Wenjie Dong, Lei Zhao, Shiyang Zhang, Shijie Zhang, Hongyun Si

**Affiliations:** 1 Department of Stomatology, The Second Affiliated Hospital of Mudanjiang Medical College, No. 15, Dongxiaoyun Street, Aimin District, Mudanjiang, 157000 Heilongjiang Province China; 2 Department of General Surgery, The Second Affiliated Hospital of Mudanjiang Medical College, Mudanjiang, 157000 Heilongjiang Province China

**Keywords:** Oral squamous cell carcinoma, Circ-KIAA0907, MiR-96-5p, UNC13C

## Abstract

**Background:**

Circular RNA (circRNA) plays an important role in regulating cell biological function and has been shown to be involved in cancer progression, including oral squamous cell carcinoma (OSCC). Circ-KIAA0907 has been found to play an anti-cancer role in OSCC, so it is worth exploring more functions and new mechanisms of circ-KIAA0907 in OSCC progression.

**Methods:**

Quantitative real-time PCR (qRT-PCR) was used to detect the expression of circ-KIAA0907, microRNA (miR)-96-5p, and unc-13 homolog C (UNC13C). Transwell assay, flow cytometry, and colony formation assay were employed to measure the migration, invasion, apoptosis, and radiosensitivity of cells. Besides, glucose uptake, lactate production, and extracellular acidification rate (ECAR) were determined to evaluate the glycolysis ability of cells. Dual-luciferase reporter assay and RIP assay were performed to confirm the interactions among circ-KIAA0907, miR-96-5p, and UNC13C. And RNA pull-down assay was used to verify the binding degree of miR-96-5p to its targets. Moreover, UNC13C protein level was examined using western blot (WB) analysis. OSCC xenograft models were constructed to perform in vivo experiments.

**Results:**

Circ-KIAA0907 was a stability circRNA with lowly expression in OSCC. Overexpressed circ-KIAA0907 could inhibit migration, invasion, and glycolysis, while promoting apoptosis and radiosensitivity in OSCC cells. In the terms of mechanism, circ-KIAA0907 could sponge miR-96-5p to regulate UNC13C expression. MiR-96-5p overexpression could reverse the inhibitory effect of circ-KIAA0907 on OSCC progression, and UNC13C knockdown also could overturn the suppressive effect of miR-96-5p inhibitor on OSCC progression. Animal experiments revealed that circ-KIAA0907 could reduce the tumor growth of OSCC by regulating the miR-96-5p/UNC13C axis.

**Conclusion:**

Our study suggests that circ-KIAA0907 restrains OSCC progression via the miR-96-5p/UNC13C axis, indicating that it may be a potential target for OSCC treatment.

## Introduction

Oral squamous cell carcinoma (OSCC) mainly refers to a type of cancer that occurs in the epithelial tissue of oral mucosa [1, 2]. OSCC often has lymph node metastasis or even distant metastasis, which poses a serious threat to the lives of patients [3, 4]. At present, most of the treatments for OSCC are based on surgery, combined with radiotherapy and chemotherapy [5, 6]. However, despite advances in research and treatment, the prognosis of patients is still poor [7]. Therefore, it is urgent to explore OSCC pathogenesis and provide new ideas for OSCC treatment.

Circular RNA (circRNA) is a special type of non-coding RNA with a circular structure, which can be used as a competitive endogenous RNA (ceRNA) of microRNA (miRNA) to indirectly regulate gene transcription [8, 9]. With the in-depth research on circRNA function, more and more evidences show that circRNAs play a key role in cancer progression [10, 11]. It has been pointed out that circRNAs may serve as potential biomarkers for cancer, which provide new targets for cancer-targeted treatment [12, 13]. Hsa_circ_100533 was discovered to be downregulated in OSCC, which played a negative role in OSCC proliferation and migration by sponging miR-933 to regulate GNAS [14]. In addition, circ_100290 was upregulated in OSCC, and it could serve as a ceRNA for miR-378a to accelerate OSCC proliferation and glycolysis via promoting GLUT1 [15]. Elucidating the molecular mechanism of circRNA is expected to provide us with a deeper understanding of the pathogenesis of OSCC.

Circ_0000140 is circularized from the KIAA0907 gene, so it is also known as circ-KIAA0907. In the previous studies, circ-KIAA0907 was found to significantly inhibit the proliferation, metastasis, and glycolytic metabolism of OSCC [16, 17]. Therefore, exploring the new mechanism of circ-KIAA0907 regulating the progression of OSCC can provide new evidence for its potential as a therapeutic target for OSCC. Our study aims to investigate the more functions of circ-KIAA0907 in OSCC development and explore the new mechanism of circ-KIAA0907 mediating OSCC progression through the hypothesis of circRNA/miRNA/mRNA axis.

## Materials and methods

### Patients and samples collection

Fifty-nine OSCC patients who underwent surgical treatment were recruited from the Second Affiliated Hospital of Mudanjiang Medical College. The 59 paired OSCC cancer tissues and adjacent normal tissues were collected and stored at − 80 °C. Each patient signed informed consent for this study. Our research was authorized by the Ethics Committee of the Second Affiliated Hospital of Mudanjiang Medical College and performed in accordance with the Declaration of Helsinki.

### Cell culture

HSC6 cells and human oral keratinocyte cells (HOK) were purchased from Tongpai Biotechnology (Shanghai, China), and OECM1 cells were obtained from Crisprbio (Beijing, China). HSC6 cells were grown in DMEM medium (SH30243.01, Hyclone, Logan, UT, USA), while HOK and OECM1 cells were cultured in RPMI-1640 medium (SH30809.01, Hyclone). An additional 10% fetal bovine serum (FBS; SH30070.03, Hyclone) and 1% penicillin/streptomycin (15140122, Invitrogen, Carlsbad, CA, USA) were added to the culture medium. All cells were plated in an atmosphere at 37 °C with 5% CO_2_.

### Quantitative real-time PCR (qRT-PCR)

Total RNA was extracted from tissues and cells with TRIzol Reagent (15596018, Invitrogen) followed by synthesizing cDNA using SuperScript Reverse Transcriptase (18064014, Invitrogen). PCR was performed using SYBR Premix Ex Taq Kit (RR420A, Takara, Dalian, China). In this, β-actin or U6 was used as internal reference. Primer sequences were exhibited in Table 1. Data were analyzed using the 2^−ΔΔCT^ method.

**Table 1:** The primer sequences in this study

	Sequences (5′-3′)
circ-KIAA0907	F: TCCCTACGGAGTACCAAGCA
	R: TGAACATAATGCATCTGTCCAGT
linar-KIAA0907	F: CATGCTCATGGCAAAAGGGAA
	R: TCTCGTCTTGAGTCTGTCCTC
miR-96-5p	F: ATGCTTTCTCAACTTGTTGG
	R: TCACCGCTCTTGGCCGTCACA
UNC13C	F: GAGTATCGTCAGCAGAAAAAGGA
	R: CTCAGTGGATAAGTTGTGAGTGG
β-actin	F: TGGATCAGCAAGCAGGAGTA
	R: TCGGCCACATTGTGAACTTT
U6	F: CTCGCTTCGGCAGCACA R: AACGCTTCACGAATTTGCGT

### Subcellular localization assay

PARIS Kit (AM1921, Invitrogen) was employed to extract the cytoplasm and nucleus RNAs of HSC6 and OECM1 cells. After that, the expression of circ-KIAA0907 in cell cytoplasm and nucleus RNAs was measured by qRT-PCR. Additionally, β-actin expression was detected as cytoplasm control, and U6 expression was measured as nucleus control.

### The identification of circRNA

Circ-KIAA0907 and linear-KIAA0907 were amplified with random primers or oligo(dT)_18_ primers. Then, qRT-PCR was performed to examine circ-KIAA0907 and linear-KIAA0907 expression. For Actinomycin D (ActD) assay, HSC6 and OECM1 cells were treated with ActD (AAT-17505, AAT Bioquest, Sunnyvale, CA, USA) for 1 h. After the cells were incubated for indicated times, the expression of circ-KIAA0907 and linear-KIAA0907 was measured by qRT-PCR.

### Cell transfection

Circ-KIAA0907 overexpression vector (circ-KIAA0907), miR-96-5p mimic and inhibitor (miR-96-5p and in-miR-96-5p), the small interfering RNA (siRNA) of circ-KIAA0907 and unc-13 homolog C (UNC13C) (si-circ-KIAA0907 and si-UNC13C), and their corresponding negative controls (pCD5-ciR, miR-con, in-miR-con, and si-con) were constructed by Ribobio (Guangzhou, China). Lipofectamine 3000 Reagent (L3000015, Invitrogen) was used to transfect them into HSC6 and OECM1 cells when the cells reached 60% confluences.

### Transwell assay

HSC6 and OECM1 cells were suspended with serum-free medium and then seeded into the upper chambers of transwell plates (BD Biosciences, Franklin Lakes, NJ, USA), in which pre-coated with Matrigel for invasion assay and non-coated for migration assay. Serum medium was added into the lower chambers. After 24 h, the cells migrated and invaded into the lower surface were fixed by methanol (B010222, Sparta Chemical Co., Ltd., Dongguan, China) and stained by crystal violet (B26890, Yuanye Biotechnology, Shanghai, China). Under a microscope (100 ×), cells were photographed and cell numbers were counted.

### Measurement of glucose uptake and lactate production

Glucose Uptake Assay Kit (KA4086) and Lactate Assay Kit (KA0833) were obtained from Abnova (Taiwan, China). Basing on the kit instructions, the glucose uptake and lactate production of HSC6 and OECM1 cells were determined.

### Cell glycolysis ability

Seahorse XFe Extracellular Flux Analyzer (Seahorse Bioscience, North Billerica, MA, USA) was performed to assess the extracellular acidification rate (ECAR) of cells to evaluate cell glycolysis. Briefly, HSC6 and OECM1 cells were seeded into the cell-culture dish. According to the instructions of Glycolysis Stress Test Kit (103020-100, Agilent, Santa Clara, CA, USA), Glucose, oligomycin, and 2-DG were sequentially added into each well. The ECAR of cells was analyzed through the Seahorse XFe Wave software (Seahorse Bioscience).

### Flow cytometry

Cell apoptosis was measured by Annexin V-FITC/propidium iodide (PI) Apoptosis Kit (70-AP101-100, MultiSciences, Hangzhou, China). In brief, HSC6 and OECM1 cells (1 × 10^6^) were harvested and washed with PBS (C0221A, Beyotime, Shanghai, China). The cells were resuspended with 1 × Binding Buffer, and then stained with Annexin V-FITC and PI in the dark for 15 min. Cell apoptosis rate was analyzed by flow cytometry.

### Colony formation assay

HSC6 and OECM1 cells were seeded into 6-well plates and irradiated with X-ray at different radiation doses (0, 2, 4, 6, and 8 Gy). After cultured for 2 weeks, the cells were stained by crystal violet. The survival fractions were calculated to assess cell radiosensitivity.

### Dual-luciferase reporter assay

The sequences of circ-KIAA0907 or UNC13C 3′UTR containing the binding sites and corresponding mutated sites of miR-96-5p were cloned into the pmirGLO vectors (Promega, Madison, WI, USA), building the wild-type (WT) and mutate-type (MUT) of circ-KIAA0907 or UNC13C 3′UTR vectors. These vectors were transfected into HSC6 and OECM1 cells with miR-96-5p mimic or inhibitor. Dual-Luciferase Reporter Assay Kit (DL101-01, Vazyme, Nanjing, China) was performed to examine cell luciferase activity after 48 h.

### RIP assay

Magna RIP Kit (17-700, Millipore, Billerica, MA, USA) was used for this assay. After transfecting with miR-96-5p mimic or miR-con for 48 h, HSC6 and OECM1 cells were collected and then lysed with RIP buffer. The cell lysates were then hatched with magnetic beads coated with Anti-Ago2 and Anti-IgG at 4 °, overnight. The immunoprecipitated RNA was extracted to detect the enrichment of circ-KIAA0907 and UNC13C using qRT-PCR.

### RNA pull-down assay

Biotin-labeled miR-96-5p (Bio-miR-96-5p) probe and negative control probe (Bio-miR-con) were transfected into HSC6 and OECM1 cells. After 48 h, the cells were lysed and then cultured with Dynabeads M-280 Streptavidin (11205D, Invitrogen). Afterwards, the bounded RNA was extracted, and the mRNA enrichment of targets was detected by qRT-PCR.

### Western blot (WB) analysis

Total proteins were extracted by RIPA Lysis Buffer (P0013C, Beyotime). The protein samples were separated by 10% SDS-PAGE gel and then transferred onto PVDF membranes (FFP28, Beyotime). After blocking with fat-free milk, the membranes were incubated with anti-UNC13C (ab234798, 1:200, Abcam, Cambridge, MA, USA) or anti-β-actin (ab8227, 1:5000, Abcam) followed by hatching with Goat anti-Rabbit (ab205718, 1:50,000, Abcam). The protein bands were developed using ECL Plus Chemiluminescence Reagent (P1050, Applygen, Beijing, China), and Image J software (NIH, Bethesda, MD, USA) was used for data analysis.

### OSCC xenograft models

Animal experiments were approved by the Animal Ethics Committee of the Second Affiliated Hospital of Mudanjiang Medical College and performed according to the Guide for the Care and Use of Laboratory Animals. Male BALB/c nude mice (*n* = 18) (Vital River, Beijing China) were divided into 3 groups (*n* = 6/group). HSC6 cells transfected with circ-KIAA0907 overexpression vector or pCD5-ciR were subcutaneously injected into mice. Non-transfected HSC6 cells were used as blank control (Empty). The tumor volume was measured every week by measuring tumor length and width. After 4 weeks, all mice were euthanatized and the tumor was removed for detecting circ-KIAA0907, miR-96-5p and UNC13C expression.

### Statistical analysis

Each experiment was performed at least 3 times, and all values were presented as mean ± standard deviation. The differences between groups were analyzed using Student’s *t* test and one-way analyses of variance followed by Tukey post hoc test. The correlations among circ-KIAA0907, miR-96-5p, and UNC13C were analyzed by Pearson correlation analysis. Statistical analysis was carried out by Graphpad Prism software (La Jolla, CA, USA). *P* < 0.05 was considered statistically significant.

## Results

### Circ-KIAA0907 is a downregulated circRNA in OSCC

**Fig. 1:**
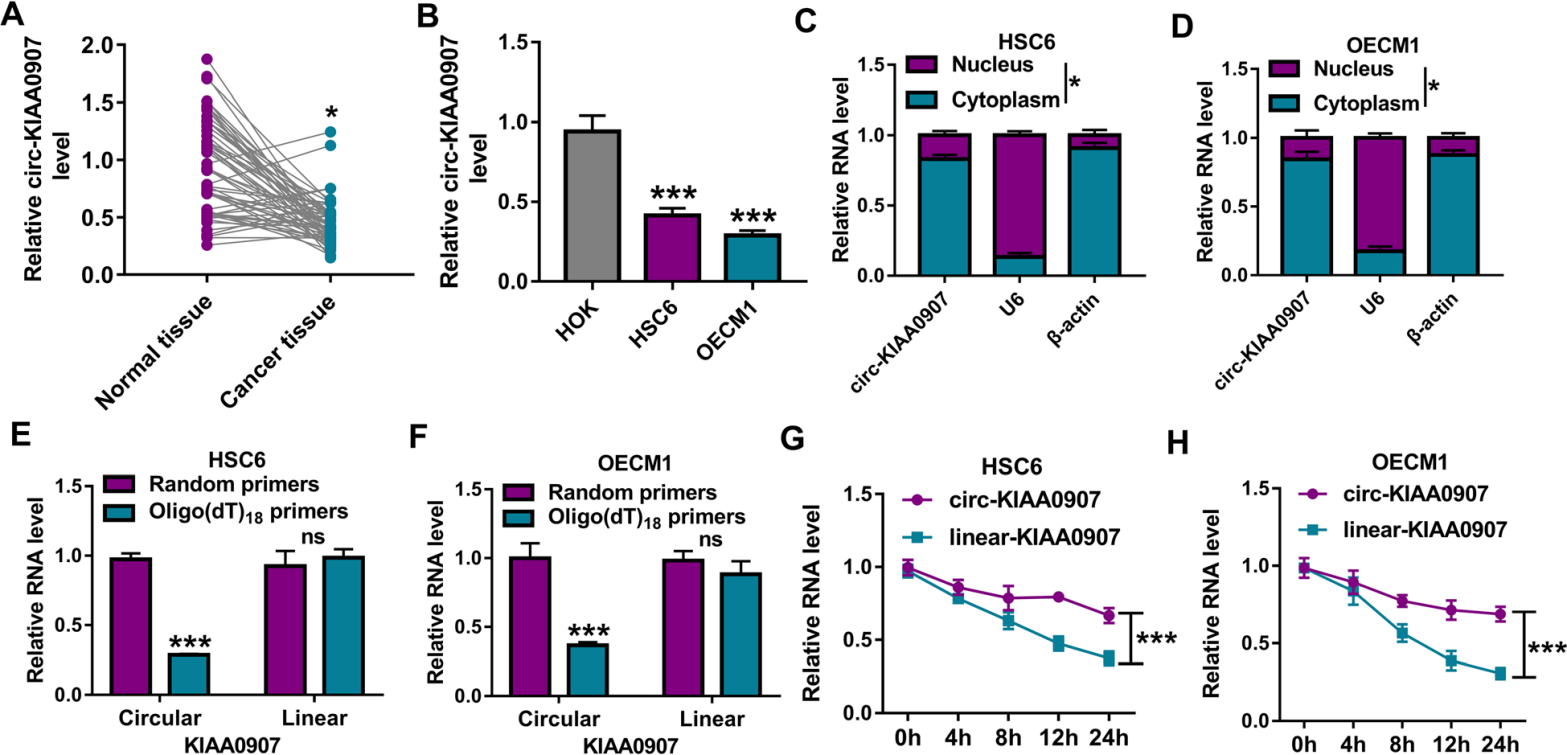
The expression of circ-KIAA0907 in OSCC tissues and cells. **a** Circ-KIAA0907 expression in 59 paired OSCC cancer tissues and adjacent normal tissues was measured by qRT-PCR. **b** QRT-PCR was used to detect the expression of circ-KIAA0907 in OSCC cells (HSC6 and OECM1) and HOK cells. **c**, **d** Subcellular localization analysis was used to confirm the localization of circ-KIAA0907 in cytoplasm and nucleus of HSC6 and OECM1 cells. **e**, **f** After amplifying with random primers and oligo(dT)_18_ primers, the expression of circ-KIAA0907 and linar-KIAA0907 was tested by qRT-PCR. **g**, **h** After the HSC6 and OECM1 cells were treated with ActD, the expression of circ-KIAA0907 and linar-KIAA0907 was assessed using qRT-PCR. *P < 0.05, ***P < 0.001

In 59 pairs of OSCC cancer tissues and adjacent normal tissues, we found that circ-KIAA0907 was significantly downregulated in OSCC cancer tissues (Fig. 1a). Moreover, circ-KIAA0907 expression also was lower in OSCC cells (HSC6 and OECM1) than that in HOK cells (Fig. 1b). Subcellular localization analysis showed that circ-KIAA0907 was mainly distributed in the cytoplasm of HSC6 and OECM1 cells (Fig. 1c, d). To determine the circular characteristics of circ-KIAA0907, we used oligo(dT)_18_ primers and random primers to amplify circ-KIAA0907 and linear-KIAA0907. By detecting the expression of circ-KIAA0907 and linear-KIAA0907 in HSC6 and OECM1 cells, we found that the relative expression of circ-KIAA0907 was markedly inhibited after amplifying oligo(dT)_18_ primers, while linear-KIAA0907 was not any changed (Fig. 1e, f), indicating that circ-KIAA0907 had not poly (A) tail. In addition, by examining the levels of circ-KIAA0907 and linear-KIAA0907 in HSC6 and OECM1 cells treated with ActD, we discovered that circ-KIAA0907 was more stable than linear-KIAA0907 (Fig. 1g, h).

### Circ-KIAA0907 suppresses the progression of OSCC

**Fig. 2:**
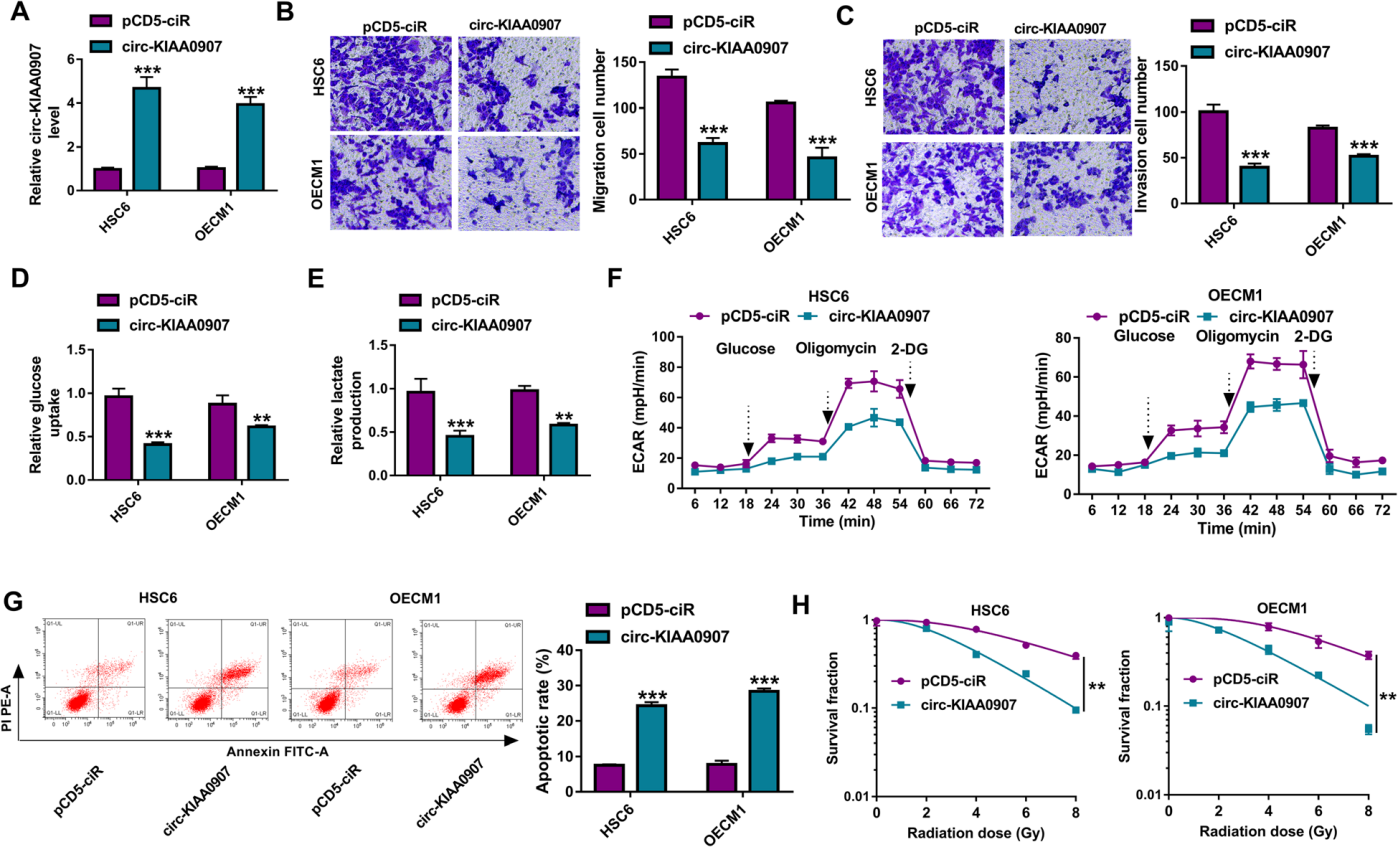
Circ-KIAA0907 suppressed the progression of OSCC. HSC6 and OECM1 cells were transfected with pCD5-ciR or circ-KIAA0907 overexpression vector. **a** The expression of circ-KIAA0907 was detected by qRT-PCR to evaluate transfection efficiency. **b**, **c** The migration and invasion cell numbers were determined using transwell assay. **d**, **f** Glucose uptake, lactate production and ECAR were measured to assess cell glycolysis. **g** Flow cytometry was used to measure cell apoptosis rate. **h** Colony formation assay was employed to analyze the radiosensitivity of cells. **P < 0.01, ***P < 0.001

To investigate the role of circ-KIAA0907 in OSCC, circ-KIAA0907 overexpression vector was transfected into HSC6 and OECM1 cells. After transfection, circ-KIAA0907 expression was remarkably enhanced (Fig. 2a). Transwell assay results showed that the migration and invasion cell numbers were notably reduced by circ-KIAA0907 overexpression in HSC6 and OECM1 cells (Fig. 2b, c). Besides, we also found that overexpressed circ-KIAA0907 could decrease the glucose uptake and lactate production of HSC6 and OECM1 cells (Fig. 2d, e). By detecting the ECAR of HSC6 and OECM1 cells, we confirmed that circ-KIAA0907 overexpression could inhibit the glycolysis ability of OSCC cells (Fig. 2f). In addition, the apoptosis of HSC6 and OECM1 cells could be promoted by circ-KIAA0907 overexpression (Fig. 2g). Colony formation assay was used to explore the influence of circ-KIAA0907 on the radiosensitivity of OSCC cells, and the results showed that the survival fractions of HSC6 and OECM1 cells were obviously decreased after upregulating circ-KIAA0907 (Fig. 2h), suggesting that circ-KIAA0907 could improve the radiosensitivity of OSCC cells.

### Circ-KIAA0907 serves as a ceRNA for miR-96-5p

**Fig. 3:**
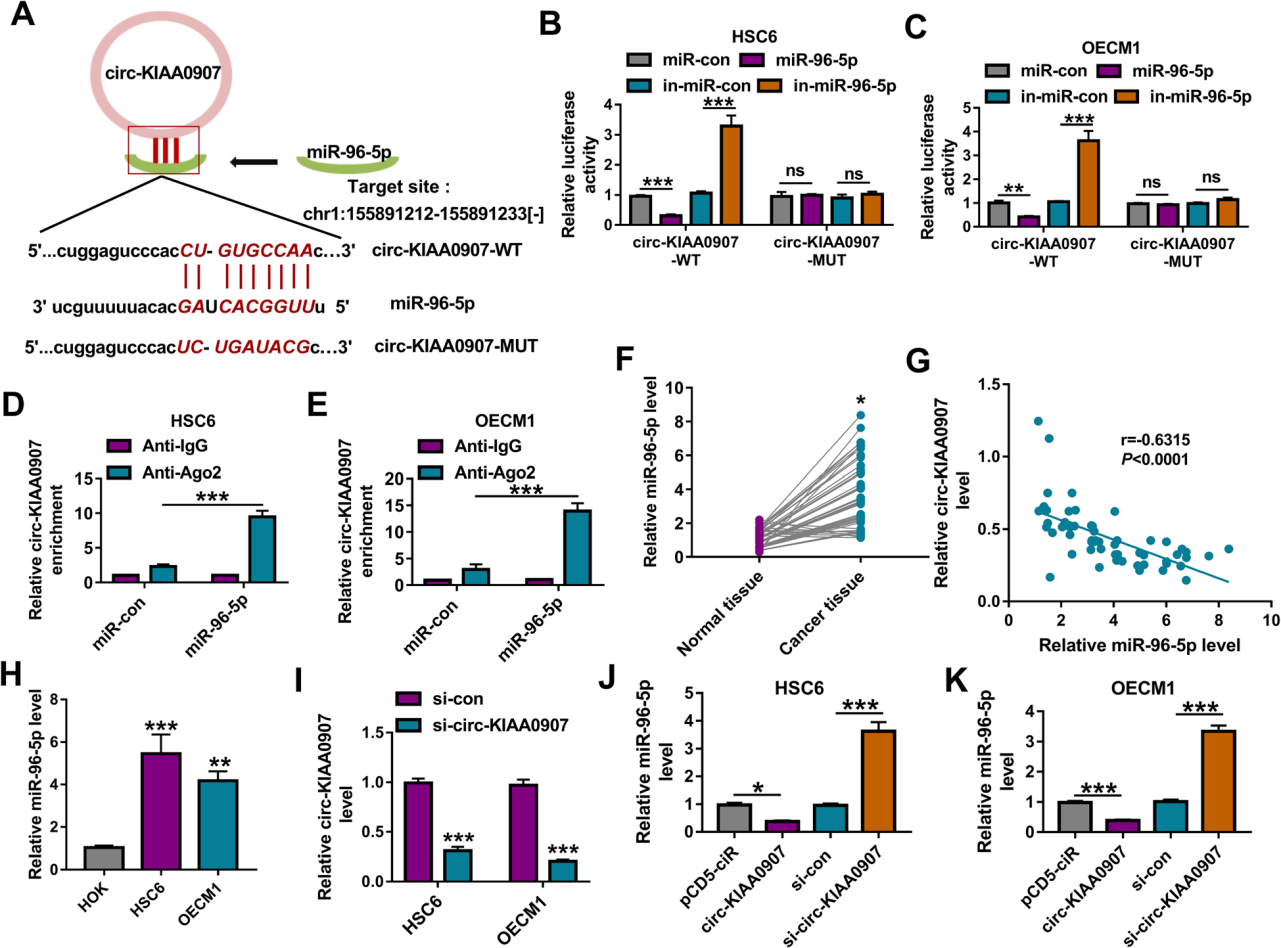
Circ-KIAA0907 sponged miR-96-5p. **a** The sequences of circ-KIAA0907 containing the miR-96-5p binding sites or mutant binding sites were shown. Dual-luciferase reporter assay **b**, **c** and RIP assay **d**, **e** were used to assess the interaction between circ-KIAA0907 and miR-96-5p. **f** The expression of miR-96-5p in 59 paired OSCC cancer tissues and adjacent normal tissues was examined by qRT-PCR. **g** Pearson correlation analysis was employed to analyze the correlation between circ-KIAA0907 and miR-96-5p expression in OSCC cancer tissues. **h** MiR-96-5p expression in OSCC cells (HSC6 and OECM1) and HOK cells was measured by qRT-PCR. **i** The transfection efficiency of si-circ-KIAA0907 was evaluated by detecting circ-KIAA0907 expression using qRT-PCR. **j**, **k** HSC6 and OECM1 cells were transfected with pCD5-ciR, circ-KIAA0907, si-con, or si-circ-KIAA0907. The expression of miR-96-5p was tested by qRT-PCR. *P < 0.05, **P < 0.01, ***P < 0.001

To illuminate the new mechanism of circ-KIAA0907 regulated OSCC progression, we performed the bioinformatics analysis. The Starbase software predicted that miR-96-5p had binding sites with circ-KIAA0907 (Fig. 3a). To further confirm the interaction between them, we performed dual-luciferase reporter assay and RIP assay. The results showed that the luciferase activity of circ-KIAA0907-WT vector could be reduced by miR-96-5p mimic and promoted by miR-96-5p inhibitor, while that of the circ-KIAA0907-MUT vector had not any changed (Fig. 3b, c). Compared to Anti-IgG control, the enrichment of circ-KIAA0907 was markedly increased in Anti-Ago2 in HSC6 and OECM1 cells transfected with miR-96-5p mimic (Fig. 3d, e). In OSCC cancer tissues, miR-96-5p was markedly upregulated and was negatively correlated with circ-KIAA0907 expression (Fig. 3f, g). Also, the expression of miR-96-5p was highly expressed in OSCC cells (HSC6 and OECM1) compared with that in HOK cells (Fig. 3h). To confirm the regulation of circ-KIAA0907 on miR-96-5p, we constructed the siRNA of circ-KIAA0907. After transfecting with si-circ-KIAA0907 into HSC6 and OECM1 cells, circ-KIAA0907 expression was remarkably decreased (Fig. 3i). Through measuring miR-96-5p expression in HSC6 and OECM1 cells transfected with circ-KIAA0907 overexpression vector or si-circ-KIAA0907, we observed that the expression of miR-96-5p was inhibited by circ-KIAA0907 overexpression, while promoted by circ-KIAA0907 knockdown (Fig. 3j, k).

### Circ-KIAA0907 regulates OSCC progression by sponging miR-96-5p

**Fig. 4:**
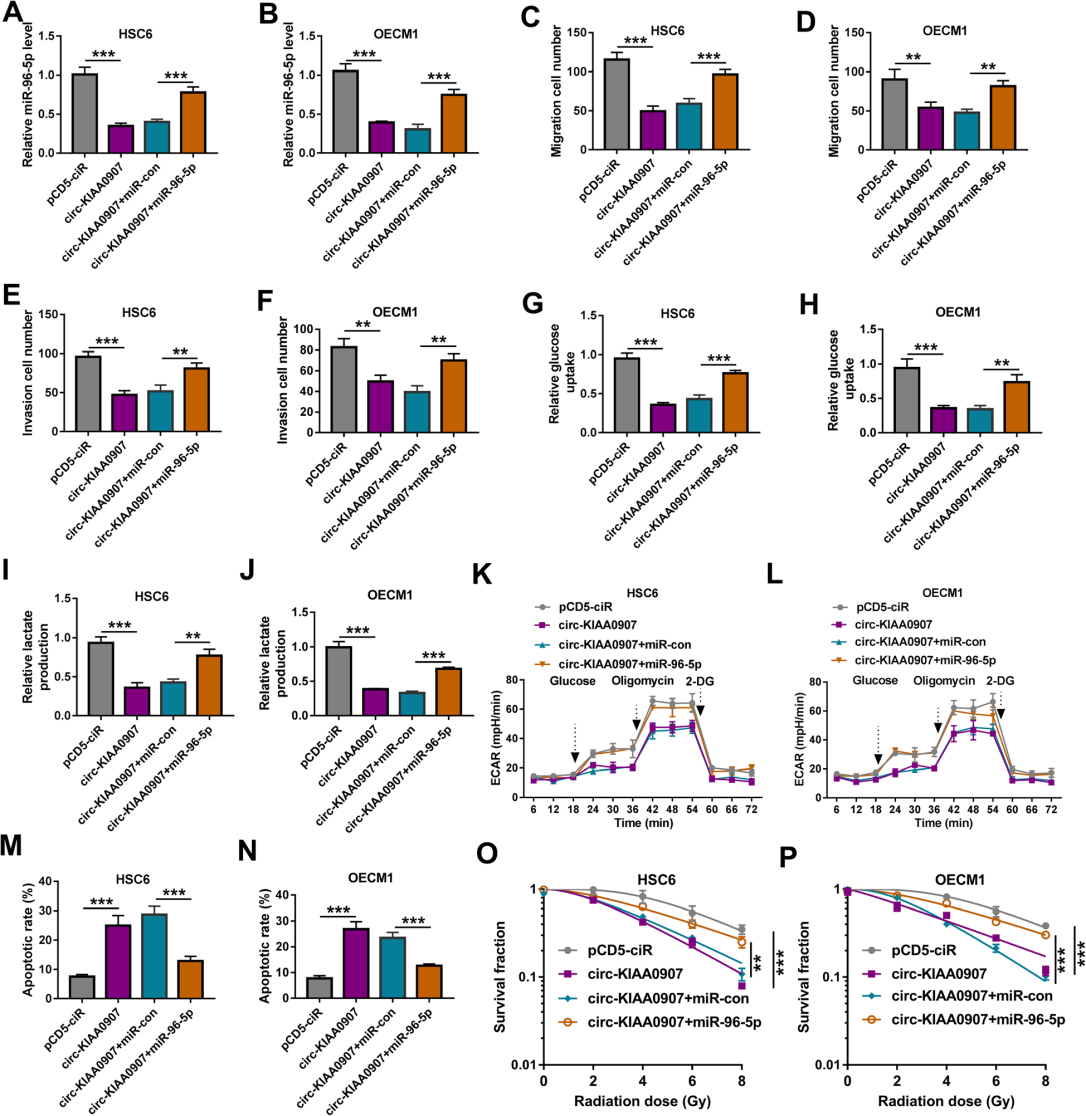
MiR-96-5p reversed the inhibition effect of circ-KIAA0907 on OSCC progression. HSC6 and OECM1 cells were transfected with pCD5-ciR, circ-KIAA0907, circ-KIAA0907 + miR-con, or circ-KIAA0907 + miR-96-5p. **a**, **b** QRT-PCR was used to examine the expression of miR-96-5p. **c**–**f** Transwell assay was employed to detect the migration and invasion cell numbers. **g**–**l** Cell glycolysis was evaluated by determining the glucose uptake, lactate production, and ECAR of cells. **m**, **n** Cell apoptosis rate was analyzed by flow cytometry. **o**, **p** The radiosensitivity of cells was measured using colony formation assay. *P < 0.05, **P < 0.01, ***P < 0.001

To further determine whether miR-96-5p participated in the regulation of circ-KIAA0907 on OSCC progression, circ-KIAA0907 overexpression vector and miR-96-5p mimic were co-transfected into HSC6 and OECM1 cells. As presented in Fig. 4a, b, miR-96-5p mimic could abolish the decreasing effect of circ-KIAA0907 on miR-96-5p expression, indicating that both transfections were successful. By assessing cell migration and invasion, we found that the suppressive effect of circ-KIAA0907 on the migration and invasion cell numbers in HSC6 and OECM1 cells could be reversed by miR-96-5p overexpression (Fig. 4c-f). Furthermore, the glucose uptake, lactate production, and glycolysis ability of HSC6 and OECM1 cells inhibited by circ-KIAA0907 also were overturned by miR-96-5p overexpression (Fig. 4g–l). Overexpressed miR-96-5p also repressed the promotion effect of circ-KIAA0907 on the apoptosis rate of HSC6 and OECM1 cells (Fig. 4m, n), and reversed the increasing effect of circ-KIAA0907 on the radiosensitivity of OSCC cells (Fig. 4o, p).

### UNC13C is a target of miR-96-5p

**Fig. 5:**
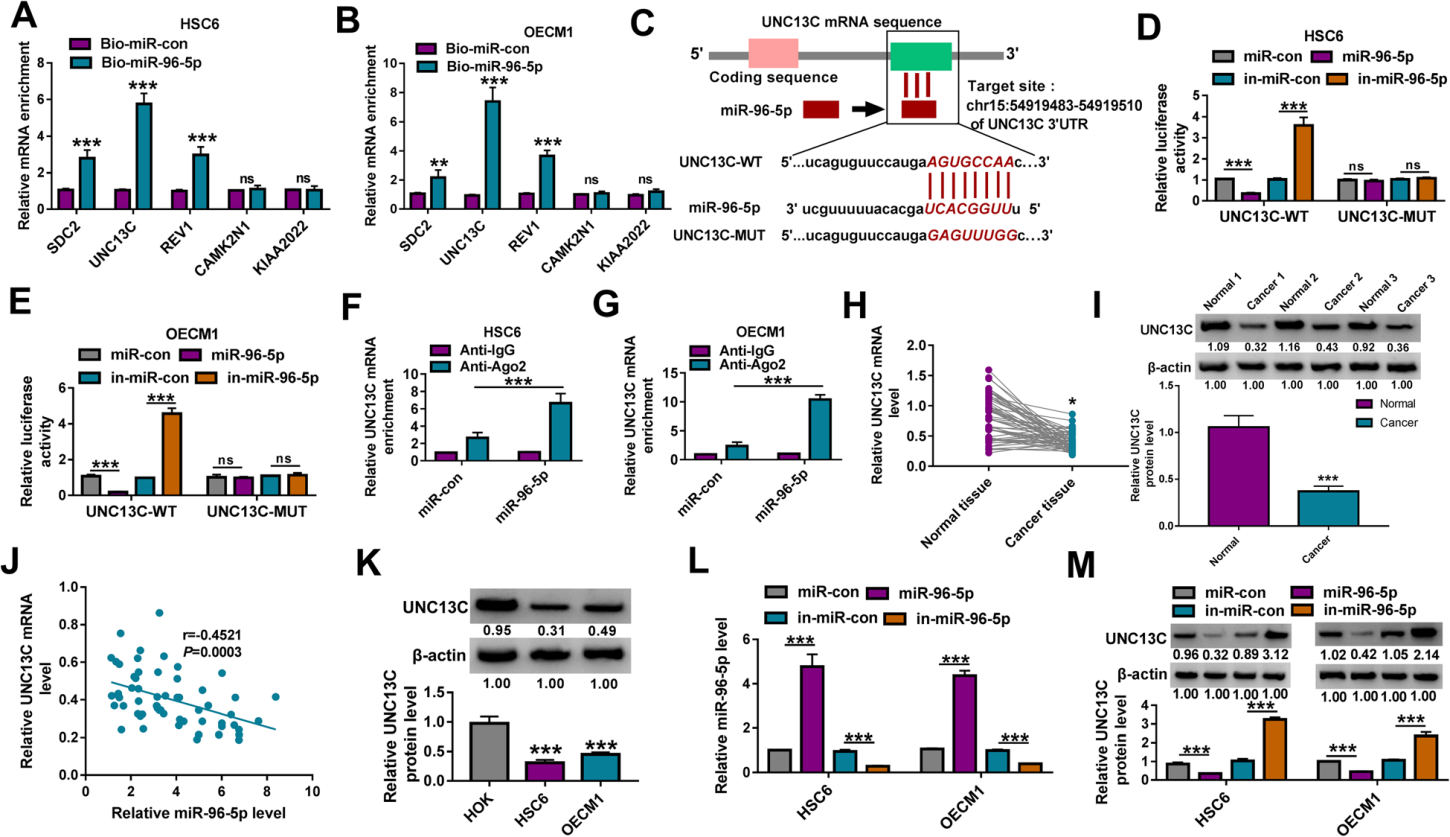
UNC13C was a target of miR-96-5p. **a**, **b** RNA pull-down assay was used to assess the binding ability of miR-96-5p and 5 target genes (SDC2, UNC13C, REV1, CAMK2N1, and KIAAA2022). **c** The binding sites and mutant sites between miR-96-5p and UNC13C 3′UTR were shown. The interaction between miR-96-5p and UNC13C was assessed by dual-luciferase reporter assay **d**, **e** and RIP assay **f**, **g**. **h** The mRNA expression of UNC13C in 59 paired OSCC cancer tissues and adjacent normal tissues was examined by qRT-PCR. **i** WB analysis was used to detect UNC13C protein expression in 3 paired OSCC cancer tissues and adjacent normal tissues. **j** The correlation between miR-96-5p and UNC13C expression in OSCC cancer tissues was assessed by Pearson correlation analysis. **k** The protein expression of UNC13C in OSCC cells (HSC6 and OECM1) and HOK cells was determined using WB analysis. **l** The transfection efficiencies of miR-96-5p mimic and inhibitor were evaluated by measuring miR-96-5p expression using qRT-PCR. **m** HSC6 and OECM1 cells were transfected with miR-con, miR-96-5p, in-miR-con, or in-miR-96-5p. The protein expression of UNC13C was detected by WB analysis. *P < 0.05, **P < 0.01, ***P < 0.001

Using the microT CDS software, we predicted the targets of miR-96-5p. According to the score, we selected the top 5 target genes (SDC2, UNC13C, REV1, CAMK2N1, and KIAAA2022). The RNA pull-down assay showed that among these target genes, the enrichment of UNC13C in the Bio-miR-96-5p probe was significantly higher than that of other target genes (Fig. 5a, b), indicating that UNC13C had the strongest binding ability to miR-96-5p. Therefore, UNC13C was selected for this study. Basing on the binding sites between them, the mutated binding sites of miR-96-5p in the 3′UTR of UNC13C were designed and the sequences were shown in Fig. 5c. Dual-luciferase reporter assay results showed that miR-96-5p overexpression could inhibit the luciferase activity of UNC13C-WT vector and its inhibitor had an opposite effect, while neither of them had any effect on the luciferase activity of UNC13C-MUT vector (Fig. 5d, e). And the RIP assay showed that UNC13C was significantly enriched in Anti-Ago2 in HSC6 and OECM1 cells overexpressed miR-96-5p (Fig. 5f, g). Besides, we discovered that UNC13C was lowly expressed in OSCC cancer tissues at the mRNA level and protein level (Fig. 5h, i). Correlation analysis suggested that the mRNA level of UNC13C was negatively correlated with miR-96-5p level in OSCC tumor tissues (Fig. 5j). In HSC6 and OECM1 cells, UNC13C protein expression also was lower than that in HOK cells (Fig. 5k). Then, miR-96-5p mimic or inhibitor was transfected into HSC6 and OECM1 cells to explore the regulation of miR-96-5p on UNC13C expression. The markedly increased and decreased miR-96-5p expression confirmed that the transfection efficiencies of miR-96-5p mimic and inhibitor were good (Fig. 5l). The detection results of UNC13C protein expression indicated that the protein expression of UNC13C could be suppressed by miR-96-5p overexpression and promoted by miR-96-5p inhibitor (Fig. 5m).

### MiR-96-5p regulates OSCC progression by targeting UNC13C

**Fig. 6:**
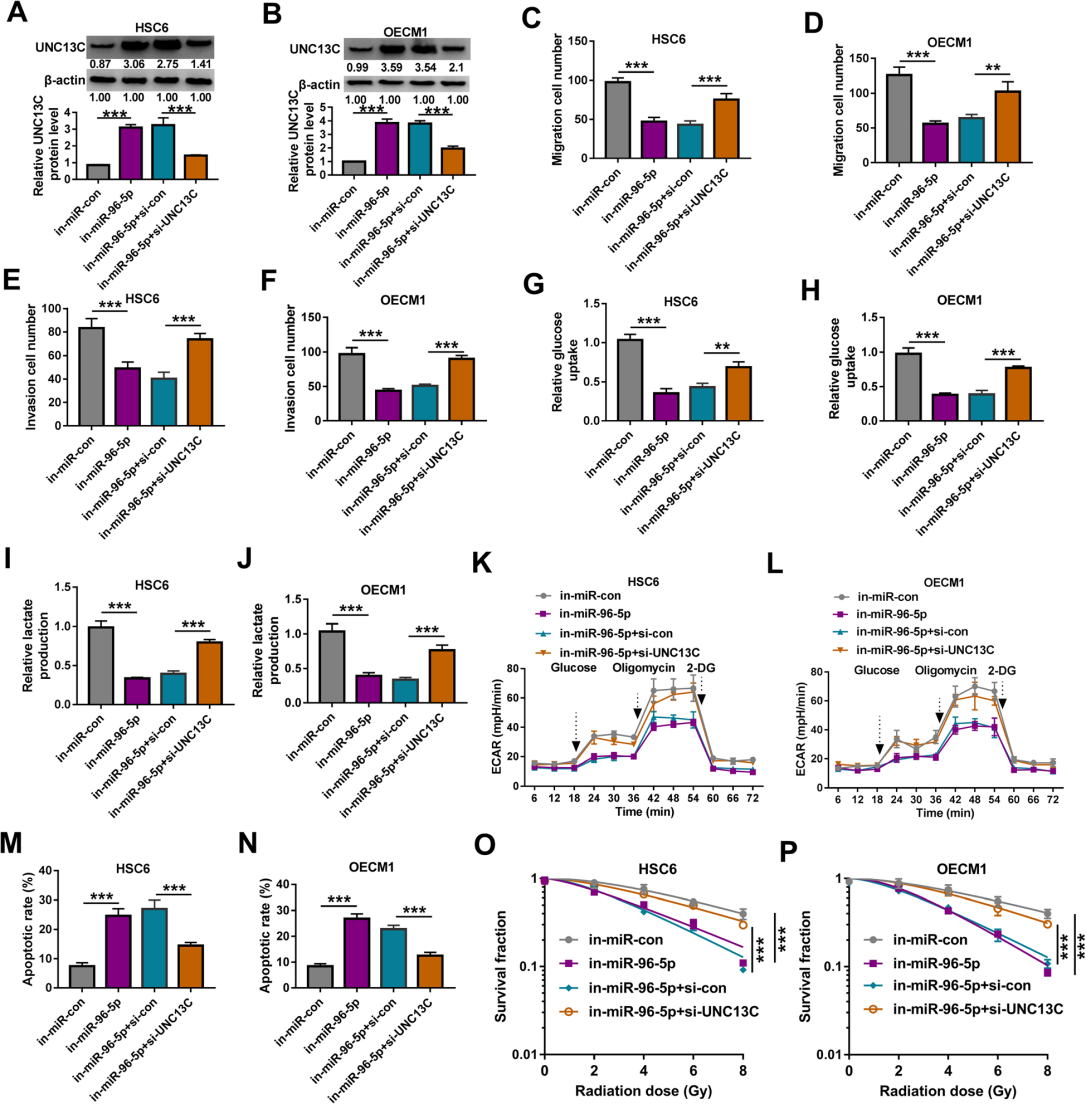
Effects of miR-96-5p inhibitor and UNC13C knockdown on OSCC progression. HSC6 and OECM1 cells were transfected with in-miR-con, in-miR-96-5p, in-miR-96-5p + si-con, or in-miR-96-5p + si-UNC13C. **a**, **b** WB analysis was used to test the protein expression of UNC13C. **c**–**f** The migration and invasion cell numbers were determined using transwell assay. **g**–**l** The glucose uptake, lactate production and ECAR of cells were detected to assess cell glycolysis. **m**, **n** Cell apoptosis rate was determined using flow cytometry. **o**, **p** Colony formation assay was performed to detect the radiosensitivity of cells. *P < 0.05, **P < 0.01, ***P < 0.001

To determine that miR-96-5p regulated OSCC progression by targeting UNC13C, in-miR-96-5p and si-UNC13C were co-transfected into HSC6 and OECM1 cells to perform the rescue experiments. The si-UNC13C could reverse the increasing effect of miR-96-5p inhibitor on UNC13C protein expression in HSC6 and OECM1 cells, confirming the effectiveness of transfection (Fig. 6a, b). Subsequently, we measured the biological functions of OSCC cells. Our data showed that miR-96-5p inhibitor could inhibit the migration and invasion of HSC6 and OECM1 cells, while this effect could be overturned by UNC13C knockdown (Fig. 6c–f). In addition, silenced UNC13C also abolished the inhibitory effect of miR-96-5p inhibitor on the glucose uptake, lactate production and glycolysis ability of HSC6 and OECM1 cells (Fig. 6g–l). Moreover, we also discovered that miR-96-5p inhibitor enhanced the apoptosis and radiosensitivity of OSCC cells, and these effects also could be reversed by UNC13C silencing (Fig. 6m–p).

### Circ-KIAA0907 sponges miR-96-5p to regulate UNC13C expression

**Fig. 7:**
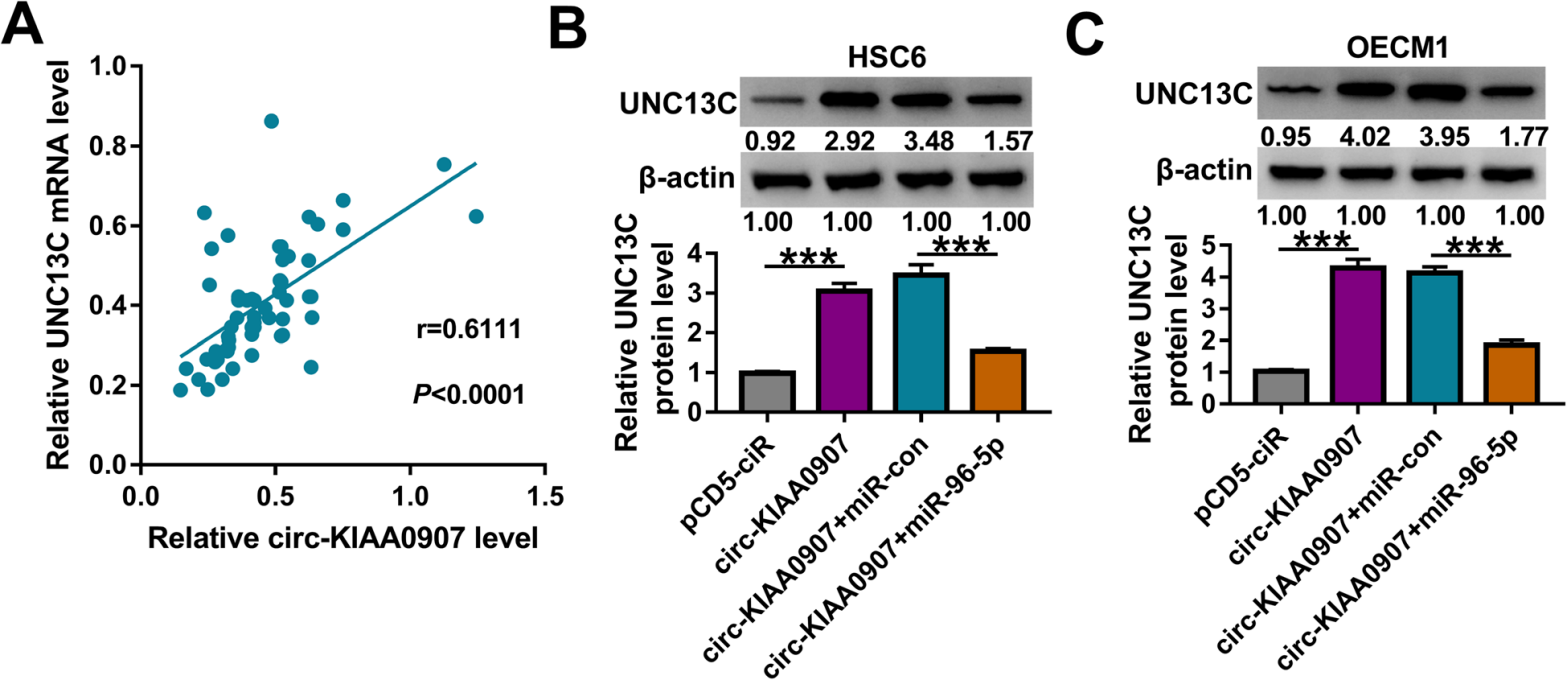
Circ-KIAA0907 regulated UNC13C by sponging miR-96-5p. **a** The correlation between UNC13C and circ-KIAA0907 expression in OSCC cancer tissues was analyzed using Pearson correlation analysis. **b**, **c** HSC6 and OECM1 cells were transfected with pCD5-ciR, circ-KIAA0907, circ-KIAA0907 + miR-con, or circ-KIAA0907 + miR-96-5p. The protein expression of UNC13C was detected using WB analysis. ***P < 0.001

In OSCC cancer tissues, we found that UNC13C mRNA expression was positively correlated with circ-KIAA0907 expression (Fig. 7a). To verify the regulation of circ-KIAA0907 on UNC13C expression, we detected UNC13C expression in HSC6 and OECM1 cells co-transfected with circ-KIAA0907 overexpression vector and miR-96-5p mimic. The detection results of UNC13C protein expression revealed that overexpressed circ-KIAA0907 could enhance UNC13C expression, while miR-96-5p overexpression could reverse this effect (Fig. 7b, c). These data revealed that circ-KIAA0907 sponged miR-96-5p to positively regulate UNC13C.

### Interference of circ-KIAA0907 inhibits OSCC tumor growth

**Fig. 8:**
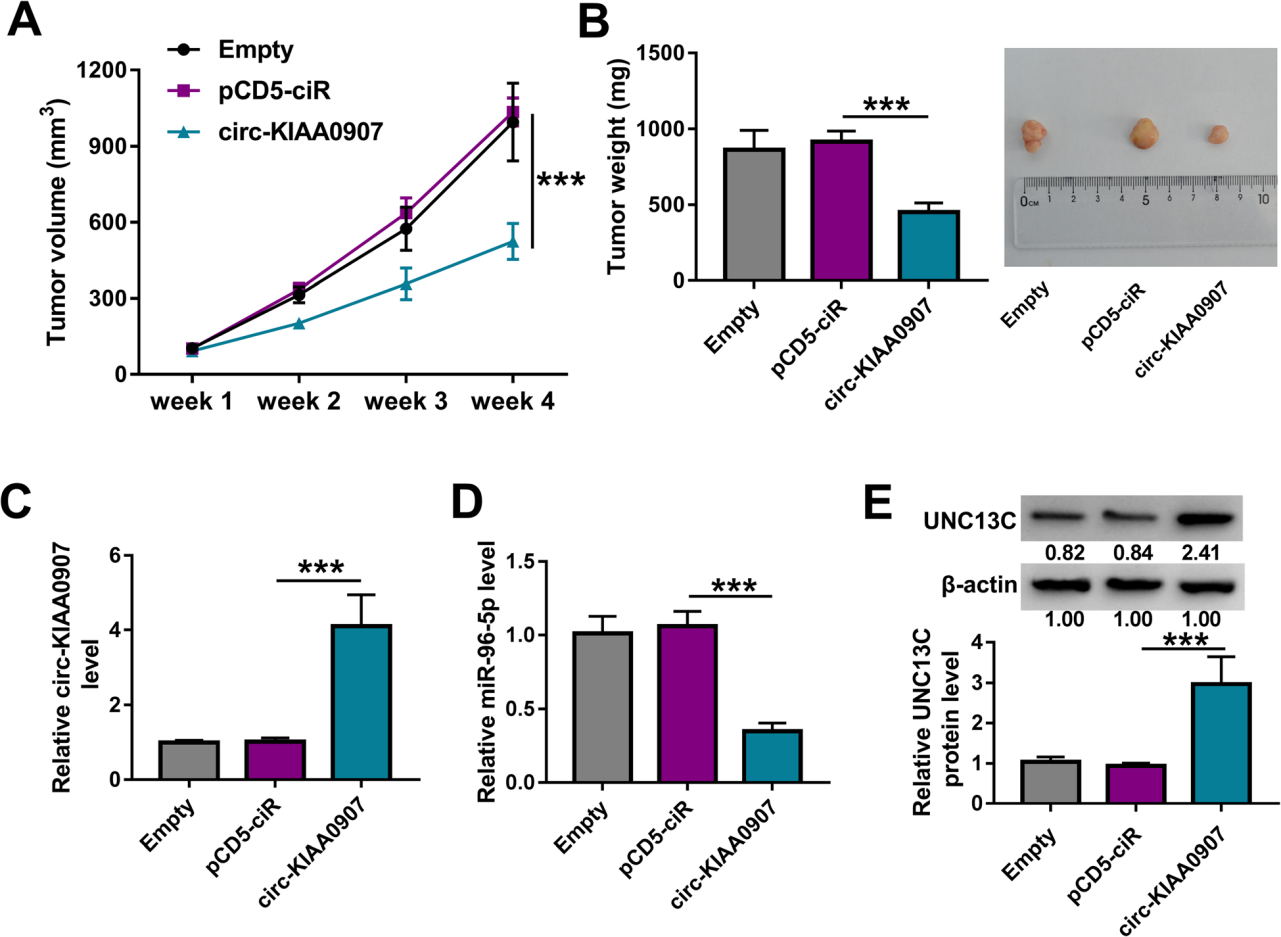
Interference of circ-KIAA0907 inhibited OSCC tumor growth. HSC6 cells transfected with pCD5-ciR or circ-KIAA0907 overexpression vector were injected in nude mice. Tumor volume **a** and tumor weight **b** were measured in mice of each group. **c**, **d** The expression of circ-KIAA0907 and miR-96-5p in the tumors of each group was assessed using qRT-PCR. **e** The protein expression of UNC13C was determined in the tumors of each group by WB analysis. ***P < 0.001

The OSCC xenograft tumor was constructed to evaluate the function of circ-KIAA0907 on OSCC tumor growth in vivo. Through measuring the tumor volume and weight, we found that compared to the control group, the tumor volume and weight of the mice in the circ-KIAA0907 group were remarkably reduced (Fig. 8a, b). In addition, the expression of circ-KIAA0907 was indeed overexpressed in the tumors of the circ-KIAA0907 group (Fig. 8c). Also, we discovered that miR-96-5p expression was significantly decreased and UNC13C protein expression was markedly enhanced in the tumors of the circ-KIAA0907 group (Fig. 8d, e).

## Discussion

The development of molecular targeted therapy provides new hope for cancer treatment [18, 19]. The discovery of useful molecular targets is crucial for the early diagnosis and late treatment of OSCC. Abnormal expression of many circRNAs has been confirmed to be closely related to OSCC development, and they are considered as potential biomarkers of OSCC, such as hsa_circ_0008309 [20], circDOCK1 [21], and hsa_circ_0004491 [22]. In the past results, Deng et al. proposed that circRNA_102459 and circRNA_043621 are differentially expressed in OSCC tissues and may be valuable biomarkers for the diagnosis of OSCC [23]. In addition, circRNA_0109291 and circRNA_002178 have been found to be significantly overexpressed in OSCC, and they can promote the progression of OSCC by regulating cell proliferation and migration [24, 25]. In contrast, circRNA_101036 has been shown to significantly inhibit the proliferation, migration, and invasion of OSCC [26]. Therefore, differentially expressed circRNAs play an important role in OSCC progression. Here, we investigated the role of circ-KIAA0907 in OSCC. Consistent with previous study results [16, 17], we confirmed that circ-KIAA0907 inhibited migration, invasion, glycolysis, and facilitated apoptosis of OSCC cells in vitro and OSCC tumor growth in vivo. Moreover, we also proposed that circ-KIAA0907 had an important positive regulatory effect on the radiosensitivity of OSCC cells. These evidences once again confirmed that circ-KIAA0907 was involved in the regulation of OSCC progression as a tumor suppressor, suggesting that circ-KIAA0907 was indeed a potential target for OSCC treatment.

The hypothesis that circRNA can be the ceRNA of miRNA has been widely confirmed [8, 9]. In previous studies, Peng et al. verified that demonstrated that circ-KIAA0907 could interact with miR-31 by dual-luciferase reporter assay [16]. Guo et al. used bioinformatics analysis to find that circ-KIAA0907 had binding sites with miR-182-5p, and confirmed that circ-KIAA0907 could sponge miR-182-5p using the dual-luciferase reporter assay and RIP assay [17]. Same as the previous method, we used bioinformatics analysis and validation of interaction relationship (dual-luciferase reporter assay and RIP assay) to indicate that circ-KIAA0907 could be used as a ceRNA of miR-96-5p. In reported studies, miR-96-5p has been shown to be involved in the regulation of cancer progression. For example, miR-96-5p could inhibit the viability and promote the chemoradiotherapy sensitivity of nasopharyngeal carcinoma by targeting CDK1 [27]. On the contrary, miR-96-5p was overexpressed in cervical cancer, which could promote cancer cell proliferation and metastasis via SFRP4 [28]. In OSCC, miR-96-5p was confirmed to promote cancer cell proliferation, invasion, and EMT process [29]. Similar to this report, our study suggested that miR-96-5p was upregulated in OSCC and it also played the pro-metastasis role in OSCC. Consistent with the previous methods [16, 17], the rescue experiments also were performed and the results showed that miR-96-5p reversed the inhibitory effect of circ-KIAA0907 on OSCC progression, indicating that miR-96-5p had a positively regulation on OSCC glycolysis, and had a negatively regulation on OSCC apoptosis and radiosensitivity. These results are a new finding, showing that circ-KIAA0907 targets miR-96-5p, a cancer-promoter, to hinder the progression of OSCC.

Through bioinformatics analysis, dual-luciferase reporter assay validation, and further rescue experiments, Wang et al. proposed that miR-96-5p targeted FOXF2 to mediate the progression of OSCC [29]. In our study, we used the same method to confirm that miR-96-5p could interact with UNC13C. UNC13C (also known as MUNc13-3), a member of the Unc/Munc family, has been found to be associated with synaptic fidelity and exocytosis [30, 31]. However, there are few studies on its role in cancer. In previous research, UCN13C was found to be abnormally expressed in gingivo-buccal OSCC [32], and another study indicated that UCN13C could inhibit OSCC proliferation and metastasis [33]. Here, the rescue experiments revealed that UNC13C silencing reversed the suppressive effect of miR-96-5p inhibitor on OSCC progression, showing that miR-96-5p targeted UNC13C to regulate OSCC progression. The anti-cancer role of UNC13C in OSCC was confirmed in our research, which was consistent with the previous study [33]. Additionally, we also confirmed that circ-KIAA0907 positively regulated UNC13C expression by targeting miR-96-5p, which improved the molecular mechanism by which circ-KIAA0907 regulated OSCC progression.

**Fig. 9:**
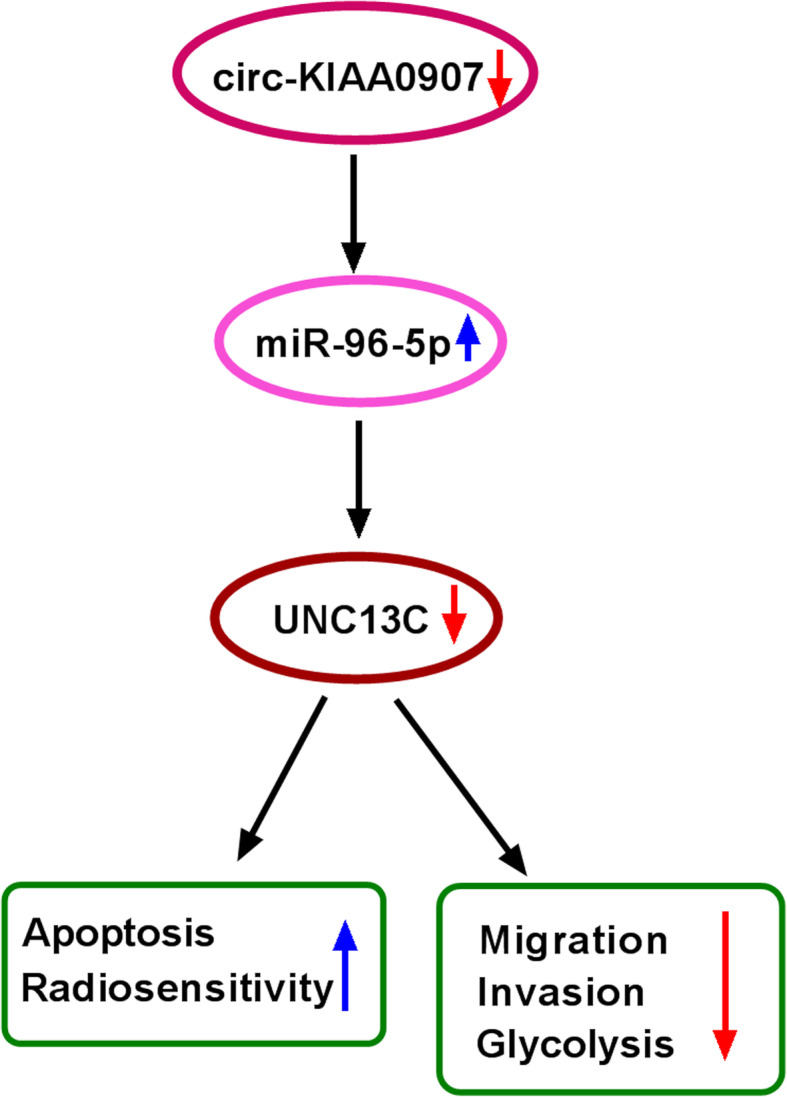
The main mechanism diagram of this research. Circ-KIAA0907 could sponge miR-96-5p to regulate UNC13C, thereby inhibiting migration, invasion, glycolysis, and promoting apoptosis and radiosensitivity in OSCC

In general, our results indicated that circ-KIAA0907 was downregulated in OSCC, and it suppressed migration, invasion, glycolysis, while enhanced apoptosis and radiosensitivity of OSCC by regulating the miR-96-5p/UNC13C axis (Fig. 9). Our results supports the conclusion that circ-KIAA0907 may be a potential target for OSCC therapy. The proposal of a new mechanism of circ-KIAA0907 regulates OSCC progression helps us deep understanding the anti-cancer role of circ-KIAA0907 in OSCC, which is of great theoretical significance.

## Data Availability

Not applicable

## Abbreviations

OSCC: Oral squamous cell carcinoma
UNC13C: unc-13 homolog C
ECAR: Extracellular acidification rate
circRNA: Circular RNA
qRT-PCR: Quantitative real-time PCR
miRNA: MicroRNA
WB: Western blot
ActD: Actinomycin D
WT: Wild-type
MUT: Mutate-type
